# Chasing a ghost: notes on the present distribution and conservation of the sooty mangabey (*Cercocebus atys*) in Guinea-Bissau, West Africa

**DOI:** 10.1007/s10329-020-00817-2

**Published:** 2020-04-21

**Authors:** Maria Joana Ferreira da Silva, Christina Paddock, Federica Gerini, Filipa Borges, Isa Aleixo-Pais, Mafalda Costa, Ivo Colmonero-Costeira, Catarina Casanova, Miguel Lecoq, Cristina Silva, Michael W. Bruford, Jorge Varanda, Tânia Minhós

**Affiliations:** 1 Organisms and Environment Division, School of Biosciences, Sir Martin Evans Building, Museum Avenue, Cardiff, CF10 3AX Wales UK; 2 CIBIO/InBio, Centro de Investigação Em Biodiversidade E Recursos Genéticos, Campus Agrário de Vairão, 4485-661 Vairão, Portugal; 3 CAPP, Centro de Administração E Políticas Públicas, Universidade de Lisboa, Rua Almerindo Lessa, 1300-663 Lisboa, Portugal; 4 Bristol Zoological Society, Clifton, Bristol, BS8 3HA UK; 5 Instituto Gulbenkian de Ciência, Rua da Quinta Grande 6, 2780-156 Oeiras, Portugal; 6 CRIA, Centre for Research in Anthropology (CRIA-FCSH/NOVA), 1069-061 Lisbon, Portugal; 7 CIAS, Centro de Investigação Em Antropologia E Saúde, University of Coimbra, Calçada Martim de Freitas, Edíficio de São Bento, 3000-456 Coimbra, Portugal; 8 Rua Barão de Sabrosa, n.º 29-1.º, 1900-087 Lisboa, Portugal; 9 Instituto Politécnico de Setúbal, Escola Superior de Tecnologia, Campus do IPS-Estefanilha, 2910-761 Setúbal, Portugal; 10 Sustainable Places Research Institute, Cardiff University, 33 Park Place, Cardiff, CF10 3BA UK; 11 Global Health and Tropical Medicine, Institute of Hygiene and Tropical Medicine (GHTM-UNL), R. da Junqueira 100, 1349-008 Lisbon, Portugal; 12 Department of Anthropology, School of Social Sciences and Humanities, Universidade Nova de Lisboa, 1069-061 Lisbon, Portugal

**Keywords:** Action plan, Gallery forest, Boé National Park, HIV-2 A, Viral jump, DNA barcoding

## Abstract

The West-African sooty mangabey (*Cercocebus atys*) is threatened by habitat loss, hunting for meat consumption, and mortality during crop-foraging events. The species’ overall demographic trend is unknown. Presence and distribution in Guinea-Bissau, a country neighbored by Senegal and Republic of Guinea, was confirmed in 1946 but the species was declared extinct in 1989 and not observed in subsequent countrywide expeditions. Narratives of its presence across southern Guinea-Bissau are scattered in reports and occurrence in the eastern part was reported in 2017, but the limits of its distribution are currently unknown. Here, we present recent geo-referenced visual and molecular-based records of the sooty mangabey for three protected areas in southern Guinea-Bissau collected as part of a region-wide survey. Individuals were observed in Cufada Lagoons Natural Park (2015) and Dulombi National Park (NP) (2016) and photographed in Boé NP (2007, 2015 and 2020). Thirty-six samples collected in Boé NP (2017) were identified as sooty mangabey using a 402 base pair fragment of the mitochondrial *cytochrome b* gene. Our work suggests a wider distribution in Guinea-Bissau than previously described, augments knowledge of the populations’ current habitat use and threats, and has implications for efforts to conserve the species in West Africa. Considering the sooty mangabey as the reservoir of the simian immunodeficiency virus that led to the human variant, HIV-2, confirmation that the Guinea-Bissau population is not extinct may lead to a better understanding of early viral jump to humans and consequent epidemic spread, specifically of the HIV-2 Subgroup A. We highlight the need for extra conservation measures by Guinea-Bissau authorities.

## Introduction

**Fig. 1:**
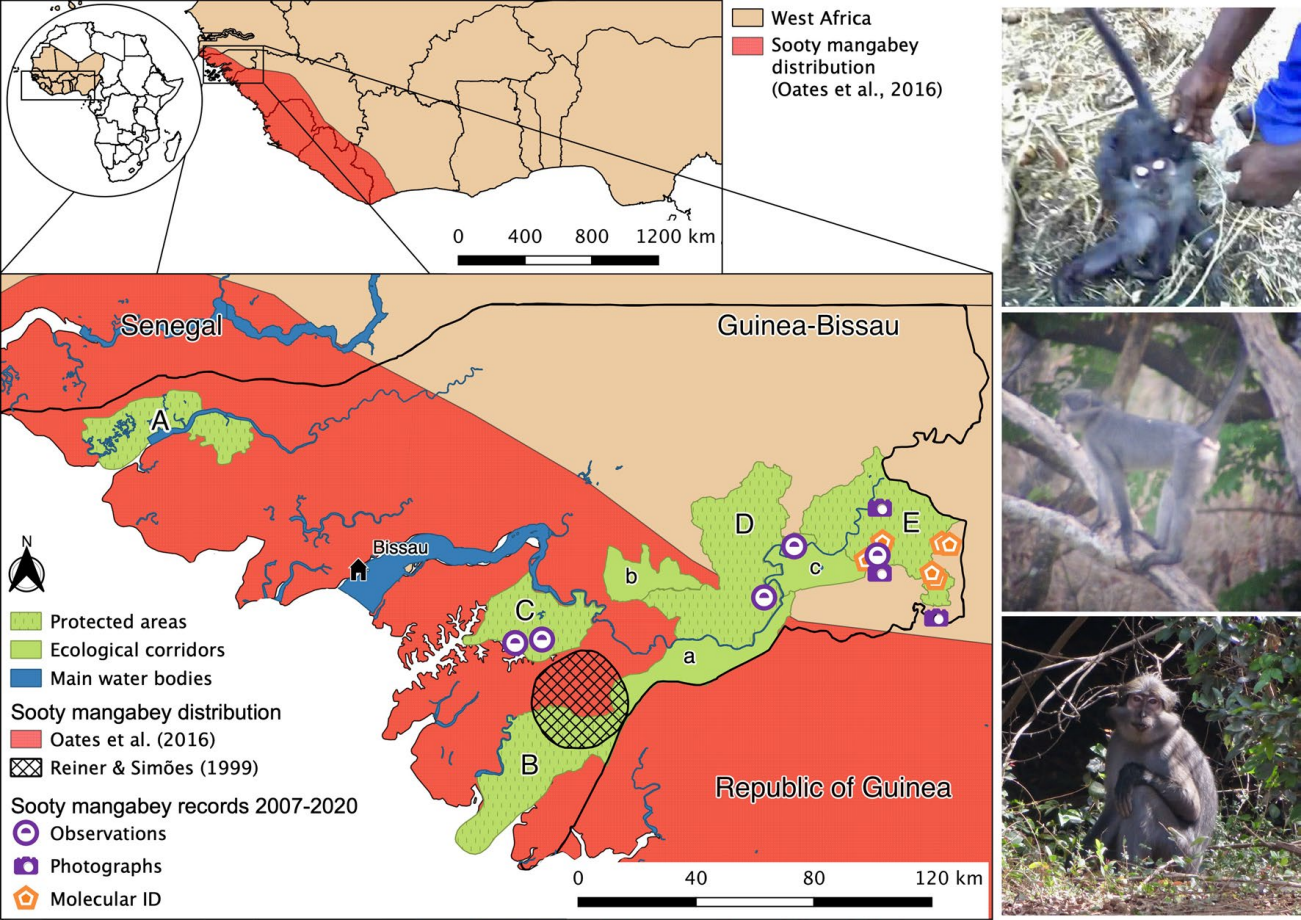
*Top left*: The distribution of the sooty mangabey in West African countries.* Bottom left*: Distribution of the sooty mangabey in Guinea-Bissau - before this work (based on Oater et al. 2016, represented in *red*, and on Reiner and Simões 1999, represented in *black crossed lines*). The geographic location of observations, photographs, and molecular records of recent presence (2007–2020) by this work are indicated. Also illustrated: protected areas and ecological corridors (*in green*) on the mainland. A: Cacheu mangroves Natural Park; B: Cantanhez National Park; C: Cufada Lagoons Natural Park; D: Dulombi National Park; E: Boé National Park, a: Cuntabane ecological corridor, b: Salifo ecological corridor and c: Tché-Tché ecological corridor). *Right, from top to bottom*: photographs taken in Boé National Park in 2007 (credits to C. S.), and in 2015 and 2020 (credits to M. L.)

The sooty mangabey (Cercopithecidae: *Cercocebus atys*; Audebert, 1797) is a terrestrial diurnal primate, native to the West African countries of Senegal, Guinea-Bissau, Guinea, Sierra Leone, Liberia, and Côte d'Ivoire (Fig. 1). The sooty mangabey occupies primary and secondary, gallery, swamp and mangrove forests, and woodland savanna (Oates et al. 2016). The species was classified as Near Threatened in 2008 by IUCN (Oates et al. 2016) and as Endangered by McGraw (2016b), and is included on Appendix II of CITES, Appendix I list of the European Union and on Class B of the African Convention on the Conservation of Nature and Natural Resources (Oates et al. 2016). Populations are thought to have been declining due to habitat loss, hunting for meat consumption, and mortality during crop-foraging events (McGraw 2016b). However, recent data on population size, distribution, and trends across the species’ range are lacking (Oates et al. 2016).

Guinea-Bissau is a small coastal country (36,125 km^2^) bordered by Senegal and Republic of Guinea, and is considered a regional stronghold for primates, with ten species reported to occur in the country (Reiner and Simões 1999; Gippoliti and Dell’Omo 2003). There are five protected areas in mainland Guinea-Bissau, four of which are located in the south (Cantanhez National Park, Cufada Lagoons Natural Park, Dulombi National Park and Boé National Park, see Fig. 1), where the majority of primates are thought to be present (Gippoliti and Dell’Omo 2003). Countrywide surveys for primates were conducted almost 20 years ago (Gippoliti and Dell’Omo 2003; Casanova and Sousa 2007; however, see Colmonero-Costeira et al. 2019) and mainly focused on species such as the western chimpanzee (*Pan troglodytes verus*)*,* the black-and-white colobus (*Colobus polykomos*, McGraw 2016a), and the Temminck’s red colobus (*Piliocolobus temminckii*, Galat-Luong et al. 2016). Recent information on primates in the northern margin of the Corubal River (such as in Dulombi National Park) is lacking, which prevents a better understanding of the direction primate conservation research should take in the region (Bersacola et al. 2018). Lack of updated information on the distribution of primates in Guinea-Bissau means the country’s Action Plan cannot be revised, and represents an important gap in conservation action plans currently being developed for primates in West-Africa (e.g., Red Colobus Conservation Action Plan: 2019–2021; Cronin et al. in prep. and the Mangadrill Conservation Action Plan; Fernández et al. 2019).

The current distribution of the sooty mangabey in Guinea-Bissau (locally known as “macaco cinzento” in creole) is uncertain. Its presence was confirmed from the 1940s onwards (Monard 1938; Frade et al. 1946) with the capture of a specimen within the current limits of the Cantanhez National Park (NP), near the border with Guinea (Frade et al. 1946). This record became the basis for the estimated distribution of the species in Reiner and Simões’s (1999) “Guinea-Bissau Wild Mammals” guide (Fig. 1). However, the species was not observed during the expeditions of Limoges (1989), Gippoliti and Dell’Omo (1996), and Gippoliti and Dell’Omo (2003) in the same region. Karibuhoye (2004) gathered reports from villagers and hunters of its occurrence in forested habitats and croplands in Cantanhez NP and Cufada Lagoon Natural Park but did not observe the species. Amador et al. (2014) reported that the sooty mangabey was considered edible by Cufada Lagoons Natural Park residents. Both studies may reflect historical records of its occurrence in the region of Cufada Lagoons Natural Park (Fig. 1). In a more recent survey in Dulombi NP, Bersacola et al. (2018) did not report the presence of the species.

Based on his results, Limoges (1989) declared the sooty mangabey to be extinct in Guinea-Bissau. However, evidence for its presence in the country after 1989 can be found in unpublished reports: Silva (2007, see Fig. 1), Goedmakers (2011), who reported its occurrence in the Boé NP, and Casanova and Sousa (2003, 2007) and Casanova (2006), who obtained evidence in the Dulombi and Quínara region and outside Cantanhez NP. Recently, Binczik et al. (2017) reported observations of individuals in secondary, semi-dense and gallery forests across the Boé region.

Evidence that the sooty mangabey is not extinct in Guinea-Bissau may represent an important route for research on the origins and transmission of the human immunodeficiency virus type 2 (HIV-2). The virus originated from the simian immunodeficiency virus found in the sooty mangabey (Visseaux et al. 2016). Recent analyses found two subtypes of HIV-2A (subtype 1 and 2) and suggested two geographically independent founding events, respectively in Ivory Coast, and in an area comprising Guinea-Bissau, Senegal, Gambia, and Guinea (Visseaux et al. 2016). Guinea-Bissau was described as the epicenter of the epidemic transmission, but the continuing narrative of extinction of the sooty mangabey in the country by Limoges (1989) (e.g., Santiago et al. 2005; Faria et al. 2002) has hindered research on the prime reservoir of the HIV-2A. Confirmation of the recent presence, and further research on sooty mangabeys in Guinea-Bissau is required to corroborate a second event of transmission of HIV-2 to humans and thus to rethink the present-day narrative on HIV-2 epidemic (Varanda 2016; in press).

Here we report visual and molecular records (photographs and fecal samples identified using DNA barcoding) that confirm recent presence (2007–2020) of sooty mangabey in Guinea-Bissau, namely in Cufada Lagoons Natural Park, Dulombi NP and Boé NP. Our work contributes to a more accurate assessment of the conservation status of the sooty mangabey, with implications for species conservation actions and for future research on the origin of epidemic HIV-2A.

## Methods

We gathered recent evidence on the presence of the sooty mangabey in Guinea-Bissau by collating photographic records (2007, 2015, and 2020) and collecting presence data (visual and/or molecular records) during expeditions to Cufada Lagoons Natural Park (December 2015), Dulombi NP (February 2016), and Boé NP (January 2017; Fig. 1). Presence and molecular data were collected as part of the PRIMACTION project (2015–2019), aimed at updating information on the presence and distribution of primate species across southern Guinea-Bissau using DNA barcoding tools. Within each national park, we visited areas reported by guards and locals to be frequently used by primates (including croplands, drinking spots, gallery, mangrove, primary and secondary forests, and savannah habitats), to collect geo-referenced visual records and non-invasive fecal material for DNA barcoding. The team members remained at those locations for a minimum of 30 min and actively searched for evidence of primate presence, such as footprints, vocalizations, and/or fecal samples.

Fecal samples were collected and preserved by the two-step method until DNA extraction (e.g*.*, Roeder et al. 2004) using silica gel Type II (S-7625, indicating for desiccation, Sigma-Aldrich® Company Ltd, Dorset, UK). We prevented contamination by exogenous DNA by wearing gloves, and head and face masks during collection of samples.

Fecal DNA extraction was carried out in dedicated facilities at *Centro de Investigação em Biodiversidade e Recursos Genéticos* (Vairão, Portugal) and at *Instituto Gulbenkian Ciência* (Oeiras, Portugal) using the Vallet et al. (2008) 2CTAB/PCI protocol with adaptations by Quéméré et al. (2010) and/or the Qiagen Stool Kit®, following the manufacturer’s protocol. A fragment of 402 base pairs (bp) of the mitochondrial *cytochrome b* gene (*cytb*) was amplified by polymerase chain reaction (PCR) using the primers GVL14724 and H15149 published by Gaubert et al. (2015). PCRs were carried out in 10 μl and included 5 μl of 1 × MyTaq™ Mix (Bioline, England), 1 μl of 10 μM primer pair mix, and 1 μl of DNA. PCRs were performed in a T100TM BIO-RAD 96-Well Thermal Cycler following cycling conditions by Gaubert et al. (2015). Successful PCR amplifications were subjected to an enzymatic clean-up by ExoSAP-ITTM PCR Product Cleanup (Exonuclease I and Shrimp Alkaline Phosphatase) by Applied Biosystems™, following the manufacturer’s protocol. Fragments were sequenced either uni or bi-directionally using the BigDye® Terminator Cycle Sequencing Kit in a 3130xl Applied Biosystems® automated sequencer. After visually correcting sequences using Geneious® v. 4.8.5 (Kearse et al. 2012), query sequences were assigned to the species by searching the NCBI database (https://www.ncbi.nlm.nih.gov/) using BLAST for the most similar voucher.

## Results

Sooty mangabeys were photographed at least three times at Boé NP, in 2007 (credits to C.S.) and in 2015 and 2020 (credits to M.L.; Fig. 1). During surveys carried out between 2015 and 2017 in protected areas in the southern part of the country, we observed the species five times, although we do not have photographs of those individuals. We observed sooty mangabeys at two localities in Cufada Lagoons Natural Park (feeding at a cropland near the village of Buba Tumbo, and in a degraded forest at the northern margin of the Buba Channel), at Dulombi NP (on the branch of a tree, near a water-spring in a gallery forest habitat) and twice in Boé NP (near the site of Pataque and on a branch of a tree on the northern bank of the Corubal River). These were full body observations (i.e., animals were not hidden by vegetation). The species was identified based on their morphology using Oates’ (2011) field guide.

We molecularly identified 36 fecal samples as sooty mangabey using DNA barcoding; they were collected in six geographically independent locations in Boé NP (approximately 3 km between sites on average, maximum distance of 29 km), surveyed on three different days. Sequences for each sample varied in length (mean sequence length average = 403.6 ± 44.7 bp; ranging between 259 and 462 bp). Only one haplotype was found. When using BLAST, the haplotype found in this study showed a 92.8, 96.8, and 99.3% identity to the three 402 bp *cytb* sequences for sooty mangabey available in the NCBI GenBank database (accession numbers KJ193498, KP090062 and NC_028592, respectively). This result indicates that the 402 bp haplotype sampled in Guinea-Bissau is between 93 to 99% identical to the nucleotide sequences deposited in NCBI GenBank identified as sooty mangabey, thus the samples harboring that haplotype can be assigned to the same species. The 402-bp nucleotide sequence reported here is available from the GenBank database under the accession number MT179319.

## Discussion

We report anecdotal observations and photographic evidence from the last 13 years (2007, 2015, 2016, 2017, and 2020) that confirm the present occurrence of sooty mangabey in Cufada Lagoons Natural Park, Dulombi NP and Boé NP, supported by the first molecular evidence of its occurrence in Boé NP. Our work definitively refutes the claim that the species is currently extinct from Guinea-Bissau. It also has direct implications for the Guinea-Bissau Action Plan, which is currently being updated and expanded to other species (the previous version, by Casanova and Sousa (2007) was for the western chimpanzees and black-and-white and red colobus), and for global conservation planning of the species, specifically the *Mangadrill Conservation Action Plan* (Fernández et al. 2019). Moreover, confirmation that the sooty mangabey is present in Guinea-Bissau may allow new routes of research in the ongoing investigation of the origin of the HIV-2A. It may also contribute, more generally, to a better understanding of the process of viral spillover (Varanda 2016; in press).

We found only one haplotype in 36 sequences collected in Boé NP, which is not unexpected given that the DNA barcoding marker used was designed to be highly conservative within the species level (Gaubert et al. 2015). Nevertheless, further study of the genetic diversity of these populations, using fast-evolving genetic markers such as the mtDNA control region and microsatellite loci, would benefit conservation action planning.

We observed and collected molecular evidence for the presence of the sooty mangabey in evergreen gallery forest habitats in Dulombi NP and Boé NP, and in croplands and degraded forest habitats in Cufada Lagoons Natural Park (Fig. 1). Our observations concur with the few previous records available for the country, such as Binczik’s (2017) report of sooty mangabeys in secondary/semi-dense forests and gallery forests in Boé NP, and Karibuhoye’s (2004) mention of crop-foraging events in maize fields by this species in Cufada Lagoons Natural Park. This suggests that mangabeys in Guinea-Bissau occupy the range of habitats observed in other locations (McGraw 2016b). Although anecdotal, these observations can help identify some threats faced by the sooty mangabey in Guinea-Bissau, which may include  habitat loss and degradation, hunting for the bushmeat trade and consumption, and for the trade of body parts for traditional medicine (Sá et al. 2012; Minhós et al. 2013). Agricultural land has been expanding for the last two decades in the country, involving the rapid replacement of slash-and-burn practices (rice, groundnuts, and sorghum) with cultivation of cash-crops, in particular cashew nuts (*Anacardium occidentale;* Catarino et al. 2015; Monteiro et al. 2017). An increase of harvested cashew nut areas occurred between 2001 and 2010 (Monteiro et al. 2017), reaching 223,000 ha of cultivated area and 130,000 tons of nut production in 2012 (data from FAOSTAT in Catarino et al. 2015). Cashew orchards are usually planted in uncultivated lands intermingled with semi-natural, tropical or savanna woodlands, even within protected areas (Hockings and Sousa 2013; Monteiro et al. 2017). The evergreen riparian forests in the Boé region, where sooty mangabeys were molecularly confirmed to be present, are threatened by periodic fires that are followed by slash-and-burn agriculture and planting of cashew orchards (Kühnert et al. 2019).

The presence of sooty mangabey individuals in a variety of habitats in Guinea-Bissau suggests a degree of behavioral and ecological flexibility that may allow populations to adapt to rapid transformation of the habitats into croplands (e.g., as observed by Wieczkowski 2005 for the Tana River mangabey *Cercocebus galeritus* in Kenya); however, the high rate of loss and degradation of primary/secondary forest habitats and higher frequency of contact between farmers and sympatric wildlife (Hockings and Sousa 2013) may further reduce and isolate populations. Narratives of mangabey fatalities during crop-foraging were documented by Karibuhoye (2004) and Amador et al. (2014), but the impact of crop-raiding mortality needs to be further assessed.

Although it is currently unknown if sooty mangabeys form interspecific associations with other nonhuman primate species in Guinea-Bissau, as observed in other locations of West Africa (e.g., Western red colobus *Piliocolobus badius* and Diana monkey *Cercopithecus diana*, McGraw 2016b), Binczik et al. (2017) and our surveys recorded mangabeys in evergreen gallery forests in Dulombi and Boé NPs, highlighting the importance of protecting these types of habitats not only for the conservation of the sooty mangabey, but also other primates, and other sympatric species.

Sooty mangabey was not found to be traded at urban primate bushmeat markets during previous molecular surveys (Minhós et al. 2013) but it may be hunted for meat consumption in Guinea-Bissau: C.S. recorded a dead individual hunted in Boé NP (Fig. 1), Karibuhoye (2004) and Amador et al. (2014) documented hunting for bushmeat consumption at Cufada Lagoons Natural Park, and the unpublished reports by Casanova and Sousa (2007) and Casanova (2006) recorded hunters of the Tombali region (where Cantanhez NP is located) mentioning sporadic hunting of mangabeys. Future research on the motivation for hunting and degree of hunting pressure on the populations of mangabeys is needed to better assess the impact of this threat.

Molecular sampling in six distinct sampling sites in the Boé region, which combined with the five geographically independent observations from Cufada Lagoons Natural Park, Dulombi NP and Boé NP between 2015–2017, and the sites in Boé NP where individuals were photographed (Fig. 1), suggests a minimum of 14 geographically-independent sightings. As our methodology was designed to survey primates in general, we cannot exclude the possibility that our results underestimate the population size of the sooty mangabey in southern Guinea-Bissau; it may be larger and more widely distributed than previously described (e.g., solely in Cantanhez NP by Frade et al. 1946; Reiner and Simões 1999). The species may have been overlooked by researchers and conservationists due to its cryptic behavior and use of lower-visibility habitats, and to a lack of systematic surveys in the country’s protected areas (but see Binczik et al. 2017). Nevertheless, we argue that extra conservation measures must be taken by Guinea-Bissau authorities to protect this species.
